# Role of NLRP3 in the pathogenesis and treatment of gout arthritis

**DOI:** 10.3389/fimmu.2023.1137822

**Published:** 2023-03-27

**Authors:** Ya-ru Liu, Jie-quan Wang, Jun Li

**Affiliations:** ^1^ Department of Pharmacy, The First Affiliated Hospital of Anhui Medical University, Hefei, China; ^2^ The Grade 3 Pharmaceutical Chemistry Laboratory, State Administration of Traditional Chinese Medicine, Hefei, China; ^3^ Department of Pharmacy, Affiliated Psychological Hospital of Anhui Medical University, Hefei, China; ^4^ Department of Pharmacy, Hefei Fourth People’s Hospital, Hefei, China; ^5^ Psychopharmacology Research Laboratory, Anhui Mental Health Center, Hefei, China; ^6^ Anhui Province Key Laboratory of Major Autoimmune Diseases, Anhui Institute of Innovative Drugs, School of Pharmacy, Anhui Medical University, Hefei, China

**Keywords:** gout arthritis, NLRP3, pathogenesis, natural products, novel compounds, ncRNAs

## Abstract

Gout arthritis (GA) is a common and curable type of inflammatory arthritis that has been attributed to a combination of genetic, environmental and metabolic factors. Chronic deposition of monosodium urate (MSU) crystals in articular and periarticular spaces as well as subsequent activation of innate immune system in the condition of persistent hyperuricemia are the core mechanisms of GA. As is well known, drugs for GA therapy primarily consists of rapidly acting anti-inflammatory agents and life-long uric acid lowering agents, and their therapeutic outcomes are far from satisfactory. Although MSU crystals in articular cartilage detected by arthrosonography or in synovial fluid found by polarization microscopy are conclusive proofs for GA, the exact molecular mechanism of NLRP3 inflammasome activation in the course of GA still remains mysterious, severely restricting the early diagnosis and therapy of GA. On the one hand, the activation of Nod-like receptor family, pyrin domain containing 3 (NLRP3) inflammasome requires nuclear factor kappa B (NF-κB)-dependent transcriptional enhancement of NLRP3, precursor (pro)-caspase-1 and pro-IL-1β, as well as the assembly of NLRP3 inflammasome complex and sustained release of inflammatory mediators and cytokines such as IL-1β, IL-18 and caspase-1. On the other hand, NLRP3 inflammasome activated by MSU crystals is particularly relevant to the initiation and progression of GA, and thus may represent a prospective diagnostic biomarker and therapeutic target. As a result, pharmacological inhibition of the assembly and activation of NLRP3 inflammasome may also be a promising avenue for GA therapy. Herein, we first introduced the functional role of NLRP3 inflammasome activation and relevant biological mechanisms in GA based on currently available evidence. Then, we systematically reviewed therapeutic strategies for targeting NLRP3 by potentially effective agents such as natural products, novel compounds and noncoding RNAs (ncRNAs) in the treatment of MSU-induced GA mouse models. In conclusion, our present review may have significant implications for the pathogenesis, diagnosis and therapy of GA.

## Introduction

Gout arthritis (GA) is one of the most common types of inflammatory arthritis, affecting an estimated 0.68% to 3.90% of adults worldwide (1). It is also described as a metabolic disorder with clinical manifestations of joint swelling, redness and pain, which caused by the deposition of monosodium urate (MSU) crystals within articular and non-articular structures in the pathological state of persistent high serum urate (2). Typically, the consequences of GA concern a series of complex complications, including impaired joint function, metabolic syndrome, diabetes, cardiovascular disease and kidney disease (3). Epidemiological studies have shown considerable regional, racial and gender differences in the prevalence of GA, with the male to female ratio ranging from 2:1 to 8:1 (4). Non-genetic factors such as increasing age, long-established unhealthy dietary, male sex and ethnic origin are potential risk factors for high serum urate concentration, which has been generally believed to be positively correlated with the occurrence and development of GA (5). Furthermore, common variations for genes involved in purine metabolism (SLC2A9, ABCG2, SLC22A11, SLC22A12, SLC17A1, SLC17A3) and NLRP3 inflammasome activation (ABRO1, ZBP1, RACK1) have been confirmed to be causally related to the course of GA (6). More recently, emerging evidence has suggested that gut microbiota may participate in the pathogenesis of GA by regulating the uric acid metabolism, inflammasome activation and immune response (7). In addition, numerous studies have demonstrated that NLRP3 inflammasome activation mediated by dysregulation of oxidative stress homeostasis may play a key role in MSU-induced GA (8, 9). However, the exact molecular mechanisms have not been fully elucidated, leading to an unfavorable diagnosis and therapy situation. Even worse, the increasing number of GA patients has imposed a huge burden to the society and family, making the breakthrough of GA diagnosis and treatment extremely urgent (10).

GA lacks specialized radical therapy and the current treatment options are mostly focused on treating acute flares and providing effective long-term maintenance (11). Anti-inflammatory drugs (e.g. non-steroidal anti-inflammatory drugs, NSAIDs) and uric acid lowering drugs (e.g. xanthine oxidase inhibitors, XOIs) have been frequently employed in the clinical treatment of GA (12). These available therapeutic drugs just alleviate symptoms or postpone the beginning of GA and even have substantial adverse reactions that may be responsible for the recurrent episodes of GA (13). The exploitation of targeted agents for GA therapy has been delayed and challenging due to the absence of reliable therapeutic targets (14). Nod-like receptor family, pyrin domain-containing 3 (NLRP3) is a special pattern recognition receptor (PRR) that functions as both a sensor and an effector, as well as being important in sterile inflammatory disorders and antimicrobial defense (15). In recent years, a great deal of studies have revealed that the pathogenic crystals can facilitate the Toll-like receptor 4 (TLR4) and nuclear factor kappa B (NF-κB)-dependent transcription of NLRP3 inflammasome components such as NLRP3, precursor (pro)-caspase-1, and pro-IL-1β, followed by the assembly and subsequent activation of NLRP3 inflammasome as well as the production of bioactive cytokines and inflammatory mediators (16, 17). Finally, the activated NLRP3 inflammasome could further trigger downstream signaling cascades involving pro-inflammatory cytokines and chemokines, and promote recruitment of neutrophils and other cells to the crystal deposit site, eventually leading to inflammatory progression and tissue destruction (18). More importantly, the dysregulated expression of NLRP3 inflammasome members and abnormal NLRP3 activity have been routinely found in GA patients and animal models (19, 20). As a consequence, it is widely speculated that the MSU-induced inflammation is mediated by aberrant activation of NLRP3 inflammasome, which plays a crucial role in the pathophysiology of GA. It also should be noted that the NLRP3 inflammasome may also represent a promising diagnostic biomarker and therapeutic target, which might aid in early diagnosis and therapy, as well as the development of therapeutic drugs.

Among the inflammasomes, the NLRP3 inflammasome is unique and the most studied because of its ability to detect a broad spectrum of significant danger signals derived from invading pathogens, such as bacterial cell wall components, bacterial RNA or the bacteria themselves, and damage-related molecular patterns (21). The NLRP3 inflammasome is assembled and activated to mediate the host’s immune response to microbial infection, whereas the chronic activation of the NLRP3 inflammasome may contribute to the pathogenesis of multiple inflammatory diseases, including GA (17). Therefore, it is crucial to block the sustained activation of NLRP3 inflammasome using some therapeutic agents, and may potentially be developed as effective avenues to treat or prevent GA (22). In this review, we first presented the background information on NLRP3 inflammasome activation. Then, we introduced the functional role of NLRP3 and relevant biological mechanisms in the pathogenesis of GA. Finally, we conducted a thorough assessment of the potential effects of therapeutic agents such as natural products, noncoding RNAs and novel compounds targeting NLRP3 on the MSU-induced GA mouse models. In conclusion, our present review will help to elucidate the involvement of NLRP3 inflammasome in the pathogenesis of GA, and may have significant implications for the development of therapeutic drugs for GA.

## Functional role of NLRP3 and relevant biological mechanisms in GA

### The activation of NLRP3 inflammasome in GA

NLRP3 belongs to NLR family members and contains an N-terminal pyrin domain (PYD), a central nucleotide binding and oligomerization (NACHT) domain and a C-terminal leucine-rich repeat (LRR) domain (23). It is well established that PYD domain may interact with the apoptosis-associated speck-like protein containing a CARD (ASC) adaptor protein, and NACHT domain can induce self-oligomerization and exert ATPase activity. Besides, LRR domain could transmit signals originating from endogenous alarmins and microbial ligands, as well as play an essential role in modulating NLRP3 activity (24). Due to the two transduction domains (PYD and CARD), ASC adaptor protein can not only interface with the upstream NLRP3, but also activate the downstream caspase-1 to perform corresponding effects (25). The NLRP3 inflammasome is a critical component of the innate immune system, and its abnormal activation is of particular relevance to the pathogenesis of a variety of inflammatory diseases (26). Specifically, a previous study has demonstrated that the expression of NLRP3, ASC and caspase-1 was significantly higher in the GA groups and hyperuricaemia groups than those in control groups (27).

The NLRP3 inflammasome is made up of a sensor, NLRP3 protein, ASC adaptor protein and pro-caspase-1, which can be significantly activated by extracellular adenosine triphosphate (ATP), certain bacterial toxins, mitochondrial damage, and various types of crystalline and particulate matter (28). Mechanistically, the NLRP3 inflammasome activation is a two-step process that requires initiation and activation signals from a variety of exogenous and endogenous activators (29). To begin, the priming signal, which is triggered by various pathogen-associated molecular patterns (PAMPs) or damage-associated molecular patterns (DAMPs), effectively promotes the transcriptional activation of NLRP3 inflammasome-containing genes such as NLRP3, pro-IL-1β, and pro-IL-18 in a TLR4/NF-κB pathway dependent manner (30). When NLRP3 activation is initially triggered, it undergoes an oligomization and then recruits ASC adaptor protein and pro-caspase-1 to assemble an NLRP3 inflammasome (24). Subsequently, the assembly and activation of NLRP3 inflammasome trigger the hydrolysis of dormant caspase-1 to active caspase-1, followed by the conversion of cytokine precursors IL-1β precursor and IL-18 precursor to mature and biologically active IL-1β and IL-18, respectively (31). Of note, it has also been suggested that neutrophil-derived serine proteases such as myeloblastin, elastase and cathepsin G, metalloproteinases and granzyme A may be responsible for IL­1β maturation, thereby contributing to the occurrence and development of GA (32). IL-1β and IL-18 belong to the IL-1 family and are proinflammatory cytokines. Functionally, mature IL-1β acts as a potent pro-inflammatory mediator in the immune system, recruiting and modulating innate immune cells to the site of infection. A substantial accumulation of IL-1β can lead to the activation of matrix-degrading enzymes that are detrimental to cartilage and bone (33). Mature IL-18 promotes interferon production and enhances cytolytic activity of natural killer cells and T cells. While the activated caspase-1 induces a proinflammatory form of pyroptosis (34). In contract, the impressive results of pharmacological inhibition of the members of IL-1 family in alleviating the symptoms of GA have also confirmed that the production of proinflammatory factors such as Il-1β and IL-18 mediated by NLRP3 inflammasome is implicated in the pathogenesis of GA (35).

**Figure 1:**
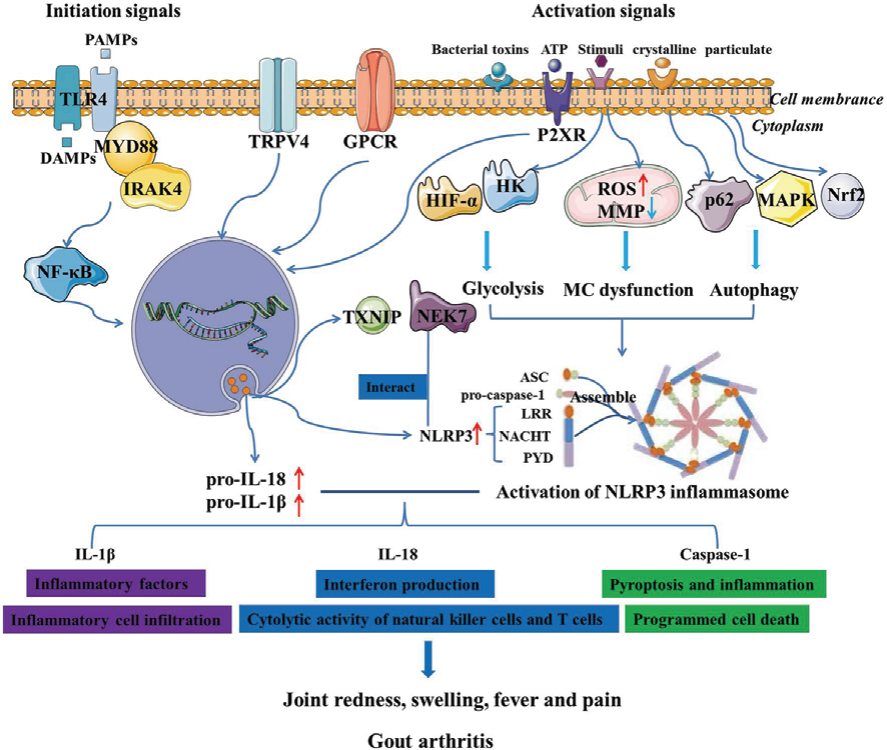
The activation of NLRP3 inflammasome and its mediated regulatory mechanisms in the pathogenesis of GA.

Despite the elaborate knowledge on the pathophysiology of GA and the activation of NLRP3 inflammasome, the functional roles of NLRP3 inflammasome and its-mediated biological mechanisms in GA remain unclear. Given that MSU crystals function as NLRP3 inflammasome agonists, it is plausible to believe that NLRP3 inflammasome plays a key role in the pathogenesis of GA (36). Autophagy, which is induced by NLRP3 inflammasome, has been demonstrated to play a dual role in NLRP3-related diseases (37). Moreover, MSU crystals are hypothesized to be involved in GA through modulating p62 accumulation, AMP-activated protein kinase (AMPK) activity and consequent activation of NLRP3 inflammasome (38). Recent studies have also found that the NLRP3-mediated multiple programmed cell death pathways participated in inflammatory response, thereby promoting occurrence and development of GA (39). When NLRP3 inflammasome is activated by MSU crystals, pro-IL-1β is cleaved by caspase-1, which promotes the pyroptosis and inflammation, thereby contributing to the pathogenesis of GA (40). It was observed that MSU crystals led to a metabolic rewiring toward the aerobic glycolysis pathway, followed by NLRP3 activation and IL-1β production, which may be closely associated with the course of GA (41). Excessive neutrophil infiltration in synovial and joint fluid is a pathogenic hallmark of GA assaults, which further resulted in joint destruction, intense pain and fever (42). Neutrophil-mediated production of IL-1β drives GA flares that is linked to the activation of the NLRP3 inflammasome in macrophages (43). MSU crystals can activate the NLRP3 inflammasome and promote the maturation of pro-IL-1β, and then induce neutrophil infiltration into joints, leading to articular swelling and pain (44). Besides, MSU crystals-induced mitochondrial damage may be responsible for the increased mitochondrial reactive oxygen species (ROS) level, NLRP3 inflammasome activation and neutrophil recruitment, implying a novel pharmacological strategy for GA prevention by blocking mitochondrial dysfunction (45). Besides, transient receptor potential vanilloid 4 (TRPV4) is a typical facilitator of MSU-induced inflammatory responses by activating NLRP3 inflammasome, and may play a pivotal role in GA (46). Purinergic signaling pathways have been shown to modulate metabolites and activate NLRP3 inflammasome through P2X ion channel receptors and G-protein-coupled receptors, stimulating IL-1β secretion and inducing GA attacks (47). Glycolytic mediators such as hypoxy-inducible factor-1α (HIF-α) and hexokinase 2 (HK) are considered to be necessary for the synthesis of NLRP3 inflammasome during inflammatory initiation and may be potential therapeutic targets for NLRP3-related diseases, including GA (48). Accordingly, Martinon et al. demonstrated that the NLRP3 inflammasome is critical for sensing MSU deposition and triggering innate immune response, and probably be a highly considerable therapeutic target for GA (49). So far, researchers have devoted increasing attention to NLRP3 inflammasome, and therapeutic strategies targeting NLRP3 inflammasome have made considerable progress in the treatment of GA (**Figure 1**).

### NLRP3 polymorphisms with susceptibility to GA

**Figure 2:**
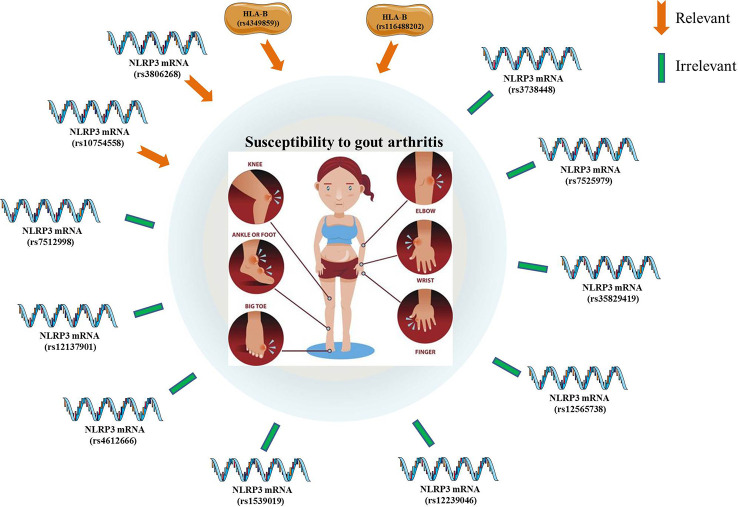
The significant associations between gout arthritis (susceptibility and potential risk) and NLRP3 polymorphisms.

A genotype-phenotype analysis of 480 primary GA patients and 480 controls found no significant association between the 17 individual SNPs of NLRP3 and the risk for primary GA (50). A case-control study of 320 GA patients and 320 controls also revealed no statistically significant relevance between the NLRP3 SNPs (rs10754558, rs7512998, and rs12137901) and the susceptibility to GA (51). An another case-control study has also confirmed no obvious correlation between NLRP3 SNPs and the potential risk for GA (52). Zhang et al. evaluated the frequency distribution of three SNPs (rs4612666, rs10754558, and rs1539019) within the NLRP3 gene in GA patients and healthy individuals. The results suggested that NLRP3 rs10754558 polymorphism may be responsible for the higher expression of components of the NLRP3/IL-1β signaling pathway, which might account for an increased susceptibility to GA in a Chinese Han population (53). Besides, it was demonstrated that the GG genotype of NLRP3 SNP (rs3806268) were correlated with an increased risk of primary GA compared to the AA genotype. In addition, there were no statistical correlations between genotyping of NLRP3 (rs4612666, rs12239046, rs10754558, rs7512998, rs12137901, rs12565738) and the potential risk of primary GA (54). Another case-control study enrolling 314 GA and 507 controls has assessed the effect of five polymorphisms of NLRP3 (rs10754558, rs35829419, rs3738448, rs3806268, and rs7525979) on the GA susceptibility. Among them, the rs3806268 AG genotype was significantly associated with decreased risk of gout, and the T-allele of rs3738448 may enhance the stability of *NLRP3* mRNA, thereby increasing the risk for GA. Bioinformatics analyses have revealed that rs3738448 and rs3806268 of NLRP3 polymorphisms may be involved in the pathophysiology of GA by regulating immune responses (55). It was reported that the rs4349859 and rs116488202 polymorphisms (within the HLA-B region) were linked to the increased risk of GA, which was no significant association with the NLRP3 polymorphisms (56) (**Figure 2**).

## Therapeutic strategies for targeting NLRP3 in GA

### Effect and mechanism of natural products in the treatment of GA

Natural products have become an important resource for the global research community to find potential GA treatment candidates (**Table 1**). *Artemisia* extract, a well-known traditional medicinal herb, is an effective antimalarial drug and has been widely used for treating various inflammatory disorders in Asian countries. Mechanistic study demonstrated that *Artemisia* extract exerts an attenuate effect on IL-1β secretion and NLRP3 inflammasome activation in bone marrow-derived macrophages (BMDMs) and MSU-induced peritonitis mouse model by suppressing the ASC oligomerization and speck formation, and might be developed as a potential therapeutic drugs for inflammasome-mediated metabolic disorders, such as GA (57). In addition, it was reported that artemisinin could inhibit NLRP3 inflammasome activation through blocking interaction between NLRP3 and NEK7, thereby reducing foot and ankle swelling in MSU-induced GA mice (58). An original study revealed that the flavonoid extract (TF) from *Selaginella moellendorffii* could down-regulate the levels of nitric oxide (NO), tumor necrosis factor alpha (TNF-α), and lactate dehydrogenase (LDH), repress ASC speck formation and NLRP3 protein expression and decrease NLRP3 inflammasome-derived IL-1β secretion and caspase-1 activation. Moreover, it was also confirmed by experiments that TF has a positive effect on alleviating paw edema and inflammatory features and preventing MSU crystals-induced GA (59). 3β,23-dihydroxy-12-ene-28-ursolic acid from *Cyclocarya paliurus* is considered to be a multi-target lead compound, which can not only reduce the expression of IL-1β, caspase-1, pro-IL-1β, pro-caspase-1, and NLRP3, but also repress NLRP3-mediated autophagy related signaling pathways. Further study revealed that it was aimed at alleviating NLRP3 inflammasome-mediated GA, which might be a potential anti-GA agent (60). *Actinidia arguta* (AA) extract significantly decreased NLRP3 inflammasome-mediated IL-1β secretion by regulating NLRP3 ubiquitination and ASC oligomerization. Also, AA extract could effectively improve MSU-induced peritonitis in a mouse model. In conclusion, AA extract exhibited an inhibitory effect on inflammasome activation *in vivo and vitro*, which substantiated the claims for its use in the treatment of inflammation and inflammation-mediated metabolic disorders, including GA (61). Ouyang et al. found that the flavonoid fraction, extracted from *Lagotis brachystachya*, could obviously attenuate hyperuricemia and GA. Specifically, the active flavonoids could not only down-regulate TNF-α and IL-1β expression levels in the joint tissue fluid of MSU-induced GA rats, but also suppress TLR4/MyD88/NF-κB pathway and NLRP3 expression in MSU-stimulated RAW264.7 cells (62).

**Table 1:** Natural products targeting NLRP3 in the treatment of GA.

Natural products	Origin of extract	Model/Have protective effect against GA?	Targets and mechanisms	References
A.princeps extract (APO)	*Artemisia princeps* var. *orientalis*	MSU-induced peritonitis and GA/Yes	Block interaction between NLRP3 and NEK7, caspase-1, and IL-1β	(57, 58)
The flavonoid extract (TF)	*Selaginella moellendorffii*	MSU-induced GA/Yes	NO, TNF-α, LDH, ASC speck formation, NLRP3, IL-1β, caspase-1	(59)
3β,23-dihydroxy-12- ene-28-ursolic acid	*Cyclocarya paliurus*	MSU-stimulated THP-1/Unvalidated	NLRP3, autophagy, IL-1β, caspase-1, pro-IL-1β, pro-caspase-1,	(60)
Actinidia arguta (AA)	*Actinidia arguta leaves*	MSU-induced peritonitis/Unvalidated	NLRP3 ubiquitination, IL-1β secretion, ASC oligomerization	(61)
The flavonoid fraction	*Lagotis brachystachya*	MSU-induced GA and MSU-stimulated RAW264.7 cells/Yes	TLR4/MyD88/NF-κB, TNF-α, NLRP3, IL-1β,	(62)
Cardamonin	*Alpinia katsumadai*	MSU-induced GA and MSU-stimulated J774A.1 cells/Yes	NLRP3, caspase-1 activation and IL-1β secretion, and COX-2 production	(63)
Cucurbitacin B (CuB)	*Cucurbitaceae*	MSU-induced GA/Yes	IL-1β, key enzymes of glycolysis, NLRP3	(64)
Coptisine	*Coptis chinensis*	MSU-induced GA and MSU-stimulated RAW264.7 cells/Yes	NLRP3 inflammasome activation	(65, 66)
Cichoric acid (CA)	*Cichorium intybus*	MSU-stimulated THP-1/Unvalidated	IL-1β, TNF-α, COX-2, PGE2, NF-Κb, NLRP3	(67)
Celastrol	*Several* spp. *of Celastraceae*	LPS-induced liver damage and MSU-induced GA/Yes	pro-caspase-1 and pro-IL-1β, NLRP3, ASC and pro-caspase-1	(68)
Curcumin	*Turmeric*	MSU-induced GA and MSU-stimulated THP-1/Yes	Inflammatory mediators (IL-1β, IL-6, TNF-α, COX-2, and PGE2), NLRP3	(69, 70)
Chaetocin	*Fungal metabolite*	MSU-induced GA/Yes	NLRP3, procaspase-1, caspase-1, pro-IL-1β, and IL-1β	(48)
β-caryophyllene	*Cloves, cinnamon, and lemon*	MSU-induced GA/Yes	NLRP3, Caspase-1, ASC, TLR4, MyD88, p65, and IL-1β	(71)
Cynarin (Cyn)	*Cynara scolymus L. and Senecio nemorensis L.*	MSU-induced GA/Yes	NF-κB and JNK pathways and NLRP3 inflammasomes	(72)
Carvacrol	*Aromatic plants*	Hyperuricemic rats/Unvalidated	ROS/NRLP3/NF-κB	(73)
Baeckein E (BF-2)	*Baeckea frutescens L.*	LPS-stimulated J774A.1 and MSU-induced GA/Yes	MAPK/NF-κB signaling pathway, NLRP3 inflammasome	(74)
Budlein A	*Viguiera robusta*	MSU-induced GA/Yes	NLRP3, Il-1β and TNF-α	(75)
Doliroside A	*Dolichos falcata Klein*	MSU-induced GA and LPS-stimulated BMDMs/Yes	caspase-1, pro-IL-1β release and NLRP3 inflammasome activation	(76)
β-Carotene	*Green plants*	MSU-induced GA and MSU-stimulated BMDMs/Yes	IL-1β, NLRP3 inflammasome	(22)
Erianin (Eri)	*Dendrobium chrysotoxum Lindl*	MSU-induced GA/Yes	NLRP3 ATPase activity/NLRP3 inflammasome assembly	(77)
Stephania hainanensis alkaloids (SHA)	*Stephania hainanensis*	MSU-induced GA/Yes	NLRP3, Caspase-1, IL-1β and NF-κB	(78)
Mollugo pentaphylla extract	*Mollugo pentaphylla*	MSU-induced GA/Yes	TNF-α, NLRP3 inflammasome and NF-κB	(79)
Gentiopicroside (GPS)	*Gentiana Macrophylla Pall*	MSU-induced GA/Yes	pro-infammatory cytokines release, neutrophil infiltration and NLRP3 inflammasome	(80)
epicatechin (EC)	*Medicinal plants and foods*	MSU-induced GA and LPS-stimulated THP-1/Yes	NLRP3 inflammasome and NF-κB signaling pathway	(81)
Epigallocatechin gallate (EGCG)	*Green tea*	MSU-challenged THP-1 and MSU-induced GA/Yes	IL-1β, IL-6, MCP-1 and serum amyloid A, NLRP3	(82)
Eucalyptol (1, 8-eucalyptol)	*Oils of eucalyptus leaves*	MSU-induced GA and MSU-stimulated RAW264.7/Yes	NLRP3 inflammasome, IL-1β and TRPV1 expression	(8)
Corilagin	*Phyllanthus urinaria*, *Phyllanthus emblica*,	LPS-stimulated BMDMs and MSU-induced GA/Yes	ROS/TXNIP/NLRP3, pyroptosis	(83)
neoastilbin	*Smilax glabra plant*	MSU-induced GA and MSU-stimulated THP-1/Yes	IL-1β, IL-6, TNF-α and NF-κB and NLRP3 inflammasome	(84)
Tetrahydropalmatine (THP)	*Corydalis yanhusuo*	MSU-induced GA/Yes	ROS-mediated NLRP3 inflammasome activation, inflammatory mediators	(85)
Wedelolactone	*Eclipta alba*	MSU-stimulated macrophages and MSU-induced GA/Yes	Ser/Thr phosphorylation of NLRP3, caspase 1 (p20) and IL-1β expression	(86)
Total glucosides of paeony (TGP)	*Paenoy roots*	MSU-stimulated THP-1/Unvalidated	MALAT1/miR-876-5p/NLRP3	(87)
Ferulic acid	*Medicinal plants and foods*	MSU-induced GA/Yes	pro-inflammatory cytokines, NLRP3 inflammasomes, caspase-1, and NF-κB	(88)
caffeic acid phenethyl ester(CAPE)	*caspase-1 activation and IL-1β production*	Primary macrophages of MSU-induced GA mouse	caspase-1 activation and IL-1β production,NLRP3 inflammasome,	(89)
Resveratrol (Res)	*Grapes, peanuts, Chinese herb Reynoutria japonica*	MSU-induced peritonitis and GA, primary BMDMs and PBMCs from GA patients/Yes	NLRP3 inflammasome, Pink1/Parkin pathway, IL-1β and NF-κB p65	(90, 91)
Rhein	*Bioactive metabolite of diacerein*	Macrophages from GA patients/Unvalidated	CASP1, NLRP3, ASC, caspase-1 and NLRP3 multiprotein complex	(92)
Phyllanthus emblica L. Extract (PEL)	*Phyllanthus emblica L.*	MSU-induced GA/Yes	NLRP3/ASC/caspase-1 TNF-α, IL-10, and IL-1β	(93)
Trans-Chalcone (1,3-diphenyl-2-propen-1-one)	*Medicinal plants and foods*	LPS-stimulated macrophages and MSU-induced GA/Yes	NLRP3, ASC, Pro-caspase-1 and Pro-IL-1β	(94)
Loganin	*Strychnos nux-vomica*	MSU-stimulated macrophages and MSU-induced GA/Yes	Assembly and activation of NLRP3 inflammasome	(45)
Leojaponin	*Leonurus japonicas*	LPS-stimulated macrophages and MSU-induced GA/Yes	NLRP3 inflammasome, RAPTOR phosphorylation	(95)
Piperine (PIP)	*Piperaceae*	MSU-induced GA/Yes	MAPK/NF-κB signaling pathway, NLRP3 inflammasome	(96)
Procyanidins procyanidin B2	*Grape seed*	MSU-stimulated macrophages and MSU-induced GA/Yes	NLRP3 inflammasome, IL-1β release, Cathepsin B	(97, 98)
Gallic acid	*Medicinal plants and foods*	MSU-induced GA/Yes	Pyroptosis, Nrf2 signaling, NLRP3 inflammasome activation	(19)
Palmatine (PAL)	*Cortex Phellodendri*	MSU-induced GA/Yes	NF-κB/NLRP3 and Nrf2 Pathways	(99)
Madecassoside	*Centella asiatica*	MSU-induced GA/Yes	IL-1β, IL-6, MCP-1and NLRP3	(100)
polysaccharide	*Isatidis Radix*	MSU-induced GA/Yes	ASC oligomerization, NLRP3, IL-1β, IL-18, IL-6, TNF-α and MPO	(101)
Dioscin	*Dioscorea*	MSU-induced GA/Yes	NLRP3, MyD88, p-IKKβ, p-p65, and NF-κB	(102)

Cardamonin, mianly found in the seeds of *Alpinia katsumadai*, possesses anti-inflammatory and antioxidative properties. A recent study showed that Cardamonin distinctly suppressed the NLRP3 expression, caspase-1 activation and IL-1β secretion, and attenuated COX-2 production in MSU-stimulated J774A.1 macrophage cells, eventually leading to the reduced synovial lining thickness and lymphocytes infiltration of GA mice (63). Cucurbitacin B (CuB), isolated from *Cucurbitaceae*, is a tetracyclic triterpene and possess a variety of biological activities. Emerging evidence suggested that CuB effectively decreased IL-1β secretion and the production of key enzymes of glycolysis in macrophages. *In vivo* studies have shown that CuB pretreatment also ameliorated foot and ankle swelling and inflammatory cell infiltration, which may be developed as a promising anti-GA agents (64). Wu et al. reported that Coptisine from *Coptis chinensis* directly inhibited caspase-1, and further blocked NLRP3 inflammasome activation. Besides, it was revealed that coptisine exhibited an attenuate effect on LPS-mediated IL-1β production and MSU-mediated mice paw edema (65). Furthermore, Berberine was demonstrated to suppress TXNIP mediated NLRP3 inflammasome activation in MSU-stimulated RAW 264.7 macrophages by activating Nrf2 anti-oxidant pathway. The *in vivo* studies also demonstrated that berberine dramatically decreased paw edema, pain score, pro-inflammatory cytokine levels and articular elastase activity, substantiating the therapeutic potential of berberine on MSU-induced GA mouse model (66). Cichoric acid (CA), isolated from *Cichorium intybus*, effectively ameliorated the MSU-induced inflammatory response in macrophages, which may be attributed to the decreased activation of NLRP3 inflammasome *via* the NF-κB signaling pathway (67). Celastrol, isolated from *several* spp. *of Celastraceae* family, is a pentacyclic triterpenoid quinonemethide and owns excellent anti-inflammatory activities. Preliminary mechanism study revealed that celastrol could interdict K63 deubiquitination of NLRP3, the formation of NLRP3 complex and the subsequent activation of NLRP3 inflammasome, thereby significantly improving the treatment of MSU-induced GA mice (68). Curcumin, the main active ingredient of turmeric, was found to markedly suppress the degradation of IκBα, the activation of NF-κB signaling pathway, and the expression levels of the their downstream genes such as IL-1β, IL-6, TNF-α, COX-2, and PGE2 in the MSU-stimulated THP-1-derived macrophages. Moreover, curcumin administration could not only protect THP-1 and RAW264.7 cells from MSU-induced mitochondrial damage, but also effectively alleviate MSU-induced paw and ankle joint swelling, inflammatory cell infiltration and MPO activity in mouse models of GA, which may be correlated with the decreased activity of NLRP3 inflammasome (69). In another study, Li et al. found that curcumin has a favorable effect on ameliorating the symptoms of MSU-induced GA. They further indicated that the therapeutic effect of curcumin on GA was mediated by the inhibition of NLRP3 inflammasome activation and NF-κB signaling pathways (70). Chaetocin is a fungal metabolite with potential anti-inflammatory activity and has recently been found to ameliorate MSU-induced GA, such as reducing local swelling and inflammatory cell infiltration. Further mechanism investigation revealed that chaetocin could decrease MSU-induced IL-1β secretion, HIF-1α expression and NLRP3 inflammasome activation. The authors also proposed a promising hypothesis that the inhibition of glycolysis pathways may be developed as a new avenue for the treatment of metabolic disease, especially GA (48). β-caryophyllene, a natural bicyclic sesquiterpene, is mainly found in natural plants and possesses embrace biological activities. An original research showed that β-caryophyllene significantly ameliorated MSU-induced inflammation and renewed ankle joints’ function, which may be mediated by the decreased expressions of NLRP3, caspase-1, ASC, TLR4, MyD88, p65, and IL-1β in the synovial tissue (71). Cynarin (Cyn), isolated from *Cynara scolymus L.* and *Senecio nemorensis L.*, is clinically applied to treat a variety of diseases caused by liver insufficiency. Based on Traditional Chinese Medicine Systems Pharmacology (TCMSP) database, literature search and qRT-PCR, Cyn was found to exert anti-inflammatory and anti-swelling effects in GA mice by inhibiting the activation of NF-κB and JNK pathways and NLRP3 inflammasomes, which may be served as a potential anti-GA drug (72). Carvacrol, produced by an abundant number of *aromatic plants*, is a monoterpenic phenol with a variety of biological and pharmacological properties, such as antioxidant, antibacterial, antifungal, anticancer, anti-inflammatory, hepatoprotective, spasmolytic and vasorelaxant. Riaz et al. found that carvacrol could effectively reduce inflammatory cytokines levels and alleviate hyperuricemia-induced oxidative stress and inflammation by regulating the ROS/NRLP3/NF-κB pathway, which may play an essential role in improving the symptoms of GA (73).

Baeckein E (BF-2), isolated from the aerial parts of *Baeckea frutescens L.*, has been found to exist inhibitory activity on NLRP3 inflammasome activation. It has demonstrated that BF-2 inhibited NLRP3 inflammasome activation, cell pyroptosis and IL-1β secretion in LPS-stimulated J774A.1 macrophages through blocking MAPK/NF-κB signaling pathway and mitochondrial damage mediated oxidative stress. More importantly, BF-2 could reduce ankle swelling and other symptoms in MSU-induced mouse models, which be a promising drug candidate against GA (74). Budlein A, produced by *viguiera robusta*, is a sesquiterpene lactone. The *in vivo* and *in vitro* studies have suggested that budlein A alleviated pain and inflammation in GA mouse models by reducing mechanical hypersensitivity, neutrophil recruitment, NLRP3, Il-1β and TNF-α mRNA expression (75).

Doliroside A, one of the active components of *Dolichos falcata Klein*, can not only suppress both LPS-induced priming and inflammasome activation in macrophages, but also ameliorate the symptoms of MSU-induced GA mouse. All these inhibitory effects conducted by Doliroside A may be attributed to the inhibition of caspase-1, pro-IL-1β release and NLRP3 inflammasome activation (76). A structure-based virtual screening analysis determined β-Carotene (provitamin A) as an efficient NLRP3 inhibitors. Confirmatory studies have shown the direct binding of β-Carotene to the pyrin domain (PYD) of NLRP3. Furthermore, β-Carotene reduced IL-1β secretion and attenuated MSU-induced inflammatory symptoms, which may represent a novel pharmacological strategy to combat NLRP3 inflammasome-driven diseases, including GA (22).

Erianin, extracted from *Dendrobium chrysotoxum Lindl*, possesses antipyretic and analgesic effects and has recently been reported to control progression of some diseases, including tumor angiogenesis, diabetic retinopathy and staphylococcus aureus infections. Zhang et al. found that Erianin inhibited NLRP3 inflammasome activation *in vitro and vivo* by directly interacting with NLRP3. Of note, Erianin could not only exert therapeutic effects on NLRP3-driven diseases, but also play an attenuate role in synovial fluid cells and monocytes from patients with IAV infection and GA (77). Stephania hainanensis alkaloids (SHA), extracted from the tuberous roots of *Stephania hainanensis*, has been widely used to treat inflammation, trauma, pain and fever. In a study conducted by Fan et al., the results showed that SHA alleviated ankle swelling, cytokines production and the inflammation infiltration around the ankle, thereby exhibiting an obvious therapeutic effects on MSU-induced GA. The increased expression of NLRP3, Caspase-1 and IL-1β in MSU-induced mice were partially relieved by SHA, suggesting NLRP3 inflammasome may be a promising therapeutic target (78). *Mollugo pentaphylla (MP)*, an annual herb, exhibits a wide range of biological activities. Lee et al. made an evaluation about the anti-inflammatory effect of an extract of MP (MPE) on MSU-induced GA in a mouse model. Surprisingly, MPE plyed an active role in relieving inflammatory paw edema and pain in the MSU-induced mice, which probably resulted from the inhibition of TNF-α, interleukin (IL)-1β, NLRP3 inflammasome and NF-κB (79). Gentiopicroside (GPS), separated from the root of *Gentiana Macrophylla Pall*, is an iridoid glucoside and has anti-infammatory, hepatoprotective and anti-cancer properties. The *in vivo* experiments demonstrated that GPS can significantly improve MSU-induced mechanical, thermal hyperalgesia and paw swelling. Moreover, GPS could suppress pro-infammatory cytokines release, neutrophil infiltration and NLRP3 inflammasome activation. Therefore, the effect of GPS on GA might be ascribed to the inhibition on NLRP3 infammasome (80). In another original study, the cell viability of THP-1 cells stimulated by LPS and MSU was observed to be significantly improved by epicatechin (EC) in a dose-dependent manner. Besides, EC concentration-dependently alleviated MSU-induced ankle edema, inflammatory cells infiltration and local ankle vascular congestion, which was most likely due to the inactivation of NLRP3 inflammasome and NF-κB signaling pathway (81). Epigallocatechin gallate (EGCG), a bioactive polyphenol in green tea, has been proven to own strong antioxidant activity and effectively prevented rheumatoid arthritis and osteoarthritis. The results suggested that EGCG could reduce MSU-induced neutrophil infiltration and the secretion of pro-inflammatory mediator such as IL-1β, IL-6, monocyte chemoattractant protein-1 (MCP-1) and serum amyloid A (SAA). In addition, EGCG treatment effectively inhibited MSU-induced NLRP3 inflammasome activation in THP-1 monocytes, suggesting that EGCG may have an anti-inflammatory effect on MSU-induced GA (82). Eucalyptol (1, 8-eucalyptol) is the main component of *eucalyptus leaf essential oil* and has anti-inflammatory and analgesic properties. It was demonstrated that eucalyptol alleviated mechanical ectopic displacement, ankle edema, and inflammatory cell infiltration of surrounding tissue in a dose-dependent manner. Mechanistically, eucalyptol decreased MSU-induced oxidative stress, NLRP3 inflammasome activation and proinflammatory cytokine production in RAW 264.7 cells as well as ankle tissue, which may be responsible for its anti-GA effect (8).

Corilagin attenuated MSU-induced joint swelling, IL-1β production, abated macrophage and neutrophil migration into the joint capsule. It was also confirmed that corilagin suppressed the ROS/TXNIP/NLRP3 pathway to restrain inflammasome activation and macrophage pyroptosis, which may play a key role in alleviating NLRP3-dependent GA (83). A flavonoid called neoastilbin is discovered in the rhizome of the *Smilax glabra plant* and has been shown to have anti-inflammatory properties. Specifically, neoastilbin significantly ameliorated swelling degree and histopathological injury in ankle joints of MSU-induced GA mice. In addition, neoastilbin markedly diminished the secretion of IL-1β, IL-6 and TNF-α and repressed the activation of NF-κB and NLRP3 inflammasome pathways *in vivo* and *vitro* (84). Tetrahydropalmatine (THP), the main active component of *Corydalis yanhusuo*, exhibits excellent anti-inflammatory and analgesic activities. It was revealed that THP suppressed ROS-mediated NLRP3 inflammasome activation, inflammatory mediators release and subsequent inflammatory cell infiltration, which may be helpful to attenuate pain and swelling in an MSU-induced GA mouse model (85). Wedelolactone, derived from the *Eclipta alba* leaves, has been widely applied as a traditional medicinal plant. In a research study conducted by Pan et al., the results showed that wedelolactone exhibited an obvious inhibitory effect on NLRP3 inflammasome activation, pyroptosis, IL-1β secretion and neutrophils infiltration. The study further suggested that the therapeutic effect of wedelolactone on MSU-induced peritonitis and GA is partially achieved by enhancing the PKA signaling pathway and further promoting the Ser/Thr phosphorylation of NLRP3 (86).

Total glucosides of paeony (TGP), extracted and purified from *paenoy* roots, has been identified as an effective drug for oral lichen planus due to its well-tolerance, safety, and effectiveness. Meng et al. reported that TGP alleviated MSU-induced damage and inflammatory response in THP-1 macrophages, which is mainly mediated by the regulation of MALAT1/miR-876-5p/NLRP3 axis (87). Despite detailed molecular biology experiments has been achieved, it is a pity that this finding has not been verified in MSU-induced GA mouse models. Ferulic acid, a dietary polyphenol, was demonstrated to significantly decrease the activity of elastase, lysosomal enzymes, nitric oxide, lipid peroxidation, the level of pro-inflammatory cytokines (TNF-α and IL-1β), and the mRNA expression of NLRP3 inflammasomes, caspase-1, and NF-κB p65, thereby improving MSU-induced inflammatory response in rats (88). Lee et al. proposed a novel regulatory mechanism by which caffeic acid phenethyl ester suppressed MSU crystals-induced caspase-1 activation, IL-1β production and subsequent NLRP3 inflammasome activation through blocking the interactions between NLRP3 and the adaptor protein ASC, providing a new preventive or therapeutic strategy for NLRP3-related inflammatory diseases such as GA (89). Resveratrol (Res), derived from *grapes, peanuts, and the Chinese herb Reynoutria japonica*, has been widely used to treat inflammatory diseases. There is a finding that Res substantially alleviated GA by preventing the activation of NLRP3 inflammasome and promoting mitophagy through the activation of Pink1/Parkin pathway (90). Resveratrol, which acts as a natural agonist for *SIRT1*, was first discovered in the root of white hellebore for the first time in 1940. Yang et al. found that resveratrol promoted autophagy by up-regulating SIRT1 to relieve inflammation in GA patients (91). Rhein is a widely used traditional Chinese medicine, which has been licensed for the treatment of osteoarthritis because of positive safety profile in humans. Chang et al. discovered that Rhein has the ability to reduce caspase-1 protease activity and IL-1 production by inhibiting the formation of the NLRP3 multiprotein complex, which can be further used for GA prevention and treatment (92).

It was widely acknowledged that *Phyllanthus emblica L. (PEL)* possesses several excellent pharmacological properties, including anti-inflammation and antioxidation. Through a network pharmacology study and experimental verification, Tao et al. revealed that PEL exhibited a significant protective effect on GA by significantly decreasing the expression of NLRP3 and caspase-1 in ankle synoviocytes as well as the levels of downstream inflammatory factors, such as TNF-α, IL-10, and IL-1β in serum (93). Due to the excellent anti-inflammatory and antioxidant proprieties of trans-Chalcone (1,3-diphenyl-2-propen-1-one), their protective effects on GA mice was further evaluated. It was observed that trans-Chalcone obviously suppressed MSU-induced mechanical hyperalgesia, edema and leukocyte recruitment in a dose-dependent manner. The *in vitro* study also confirmed that trans-Chalcone reduced NF-κB activation and the mRNA expression of the NLRP3 inflammasome components (NLRP3, ASC, Pro-caspase-1 and Pro-IL-1β) (94). Loganin is an iridoid glycoside compound that possesses anti-inflammatory activity. Choi et al. found that loganin alleviated MSU crystals-induced inflammation in a GA mouse model, which was accompanied by the inhibition of the assembly and activation of NLRP3 inflammasome. Meanwhile, it was also revealed that loganin has a reverse effect on MSU-induced mitochondrial damage in macrophages (45). Leojaponin, a diterpenoid compound isolated from *Leonurus japonicas*, ameliorated MSU-induced GA through the inhibition of NLRP3 inflammasome activation. The mechanism study suggested that the inhibitory effect of Leojaponin on NLRP3 inflammasome may be associated with enhanced autophagy *via* upregulating RAPTOR phosphorylation (95). Piperine (PIP), a phytochemical compound derived from *Piperaceae* family, exists a favorable anti-inflammatory activity. Based on an *in silico* study and molecular validation, it was demonstrated that PIP exerted an immunosuppression activity through the disruption of NLRP3 inflammasome, indicating the potential of PIP as a candidate agent for the treatment of GA (96). Procyanidins, derived from *grape seed*, significantly attenuated gout pain and ankle swelling in MSU-induced GA mouse model by inactivating NLRP3 inflammasome and reducing IL-1β release (97). Also, the study conducted by Qiao et al. suggested that procyanidin B2, a natural dietary compound, suppressed MSU-stimulated inflammation and immune cell infiltration in the air pouch skin and paws of GA mouse, which possibly be accomplished by the decreased expression of IL-1β, Cathepsin B and NLRP3 (98). A recent study has revealed that gallic acid could moderate MSU-induced NLRP3 inflammasome activation and pyroptosis, and restrain macrophages and neutrophils migration into joint synovitis, thereby mitigating the joint swelling. Further investigation also suggested the therapeutic potential for GA are mainly achieved by limiting NLRP3 inflammasome activation and pyroptosis *via* enhancing Nrf2 signaling (19). Palmatine (PAL), a protoberberine alkaloid, has been proven to mitigate the MSU-induced joint swelling and neutrophil infiltration *via* inhibiting NF-κB/NLRP3 and Nrf2 signaling pathways, and may represent a potential candidate for the treatment of GA (99). Madecassoside, purified from traditional Chinese medicine, exhibits potent anti-arthritic effects. The experimental study showed that Madecassoside alleviated MSU-triggered pad swelling, joint 99mTc uptake, and joint inflammation, which was accompanied with decreased expression of caspase-1, IL-1β and NLRP3 (100). The polysaccharide, extracted from *Isatidis Radix*, has a positive effect on alleviating GA. Mechanistically, polysaccharide suppressed the oligomerization of ASC and subsequently blocked the formation of NLRP3 inflammasome, which probably lead to the decreased ankle thickness and IL-1β, IL-18, IL-6, TNF-α and MPO secretion (101). Dioscin, an active ingredient, has been reported to have a protective effect against MSU-mediated inflammatory response in GA mouse model through inhibiting NLRP3 inflammasome and TLR4/NF-κB signaling pathway activation (102).

### Effect and mechanism of novel compounds in the treatment of GA

In recent years, novel synthetic compounds have also shown promising therapeutic advantages for GA through single-target and even dual-target action (**Table 2**). As follows, we summarized some of the novel compounds currently being investigated in GA, which may be used in the treatment in the future. It was suggested that 4-(2-(4-chlorophenyl)-1-((4-chlorophenyl)amino)ethyl) benzene-1,3-diol (CBED) could effectively improve the MSU-induced ankle swelling and histopathological damage. On the other hand, it was revealed that CBED may exhibited a dual inhibitory effect on XOD activity and NLRP3 inflammasome activation in a dose dependent manner (103). On the basis of bioinformatics analysis and molecular docking, it was found that dapansutrile may not only selectively target NLRP3 to attenuate the inflammatory response and pyroptosis, but also block the chemotaxis and activation of inflammatory cells by regulating IL-1β, IL6, IL17A, IL18, MMP3, CXCL8, and TNF-α (104). Overall, dapansutrile, also known as OLT1177, suppressed MSU-induced joint swelling and inflammation, and may be highly effective in ameliorating GA by functioning on NLRP3 inflammasome (105). P2Y_14_R plays a critical role in GA by regulating NLRP3-mediated pyroptosis of macrophages (116). Several P2Y_14_R antagonists have been developed, which have been found to block the activation of NLRP3 inflammasome through its inhibitory effects on P2Y_14_R-cAMP pathways. It was demonstrated that compound 8, exhibited the good antagonistic activity against P2Y_14_R, could restrain MSU-induced pyroptosis of THP-1 cells through blocking the activation of NLRP3 inflammasome (106). In another study, 2-phenyl-benzoxazole acetamide derivative (compound 52) showed a strong inhibitory effect on paw swelling and inflammatory cell infiltration through cAMP/NLRP3/GSDMD signaling pathways in MSU-induced GA mice (107). In a recent study, Lu et al. reported that 3-amide-5-aryl benzoic acid derivatives (compound 11m) played a favorable *in vivo* role in relieving MSU-induced paw swelling and inflammatory infiltration through antagonist of P2Y_14_R activity and subsequent inhibition of NLRP3 inflammasome activation (108). As a result, these compounds can be used as a good lead compound for further optimization to obtain more promising compounds for the treatment of GA. Besides, (E)-1-(6-methoxybenzo[d]oxazol-2-yl)-2-(4-methoxyphenyl)ethanone oxime (5d) has an active effect on improving the symptoms of hyperuricemia and GA. Further investigation also revealed that compound 5d can not only suppress XOD activity, but also block the activation of NLRP3 inflammasome and TLR4 signaling pathway, which may be developed as a multi-targeting candidate compound (109). A total of 22 novel benzoxazole and benzimidazole derivatives were synthesized as NLRP3-XOD dual inhibitors. Notably, compound 9b significantly decreased the swelling degree of the ankle joint induced by MSU crystals with less serious adverse reactions and better tolerance when compared with cochicine (110). Curcumin analogue AI-44 bound to cathepsin B and suppressed the NLRP3 inflammasome complex assembly, thereby reducing the recruitment of macrophages and neutrophils and alleviating the foot paw swelling and thickness. Collectively, AI-44 may be applied as a novel drug candidate for the treatment of GA (111). Cao et al. found that NSC697923, a potential caspase-1 inhibitor, effectively inhibited MSU-induced joint swelling by suppressing NLRP3 inflammasome activation and IL-1β release in a caspase-1 dependent manner, thus warranting further investigation as a candidate therapeutic drug for treating NLRP3 inflammasome-related GA (112). Hesperidin methylchalcone (HMC), (E)-1-[4-[[6-O-(6-Deoxy-α-L-mannopyranosyl)-ß-D-glucopyranosyl]oxy]-2-hydroxy-6-methoxyphenyl]-3-(3-hydroxy-4-methoxyphenyl)-2-propen-1-one, showed great bioavailability, metabolic stability and tissue distribution. It was confirmed that HMC could effectively suppress MSU-induced NF-κB activation (41%, p < 0.05), gp91^phox^ (66%, p < 0.05) and NLRP3 inflammasome components mRNA expression *in vivo* (72%, 77%, 71% and 73% for NLRP3, ASC, pro-caspase-1 and pro-IL-1 β, respectively, p < 0.05), thereby reducing the MSU-induced hyperalgesia (44%, p < 0.05), edema (54%, p < 0.05) and leukocyte infiltration in a dose-dependent manner (113). Spirodalesol, a compound produced from the ascomycete fungus *Daldinia eschscholzii*, has been shown to inhibit the activation of the NLRP3 inflammasome. Liu et al. discovered that spirodalesol analog compound 8A alleviated GA in mice by interrupting the assembly of the NLRP3 inflammasome complex and inhibiting the activation of caspase-1 *via* directly targeting adaptor protein ASC (114). Furthermore, it was reported that (N-{3-[(2-aminoethyl) sulfamoyl] phenyl}-2-methyl-3- [3-(trifluoromethyl) phenyl] propanamide hydrochloride) blocked ATP-induced activation of the NLRP3-caspase-1-IL-1β pathway and exerted promising effects in reducing joint inflammation in GA rats (115).

**Table 2:** Novel compounds targeting NLRP3 in the treatment of GA.

Compounds	Structure	Model/Have protective effect against GA?	Targets and mechanisms	References
CBED	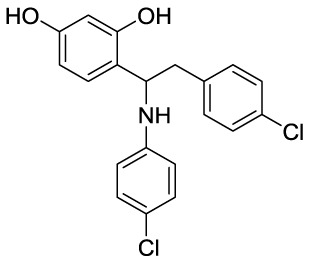	MSU-induced GA and MSU-stimulated THP-1/Yes	XOD, NLRP3, IL-1β	(103)
dapansutrile	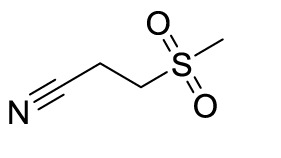	Primary macrophages of MSU-induced GA mouse and MSU-induced GA/Yes	NLRP3, IL-1β, IL-17A, IL-18, MMP3, CXCL8,TNFIL-1β, IL-6, myeloperoxidase, and CXCL1	(104, 105)
Compound 8	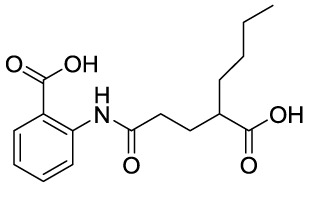	MSU-stimulated THP-1/Unvalidated	P2Y_14_R-cAMP pathways, NLRP3, pyroptosis	(106)
Compound 52	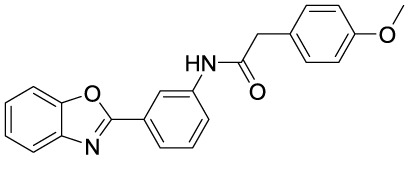	MSU-stimulated BMDMs and MSU-induced GA/Yes	P2Y_14_R-cAMP/NLRP3/ GSDMD	(107)
Compound 11m	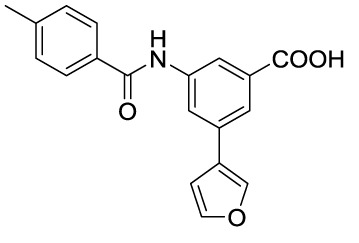	MSU-induced GA/Yes	P2Y_14_R -cAMP/NLRP3	(108)
Compound 5d	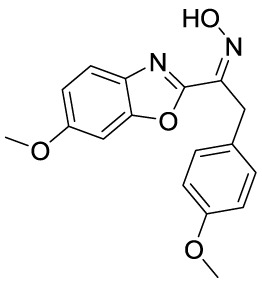	MSU-stimulated THP-1 cells and MSU-induced GA/Yes	XOD, NLRP3,TLR4	(109)
Compound 9b	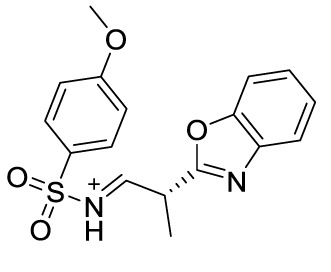	MSU-stimulated THP-1 cells and MSU-induced GA/Yes	NLRP3 and XOD	(110)
AI-44	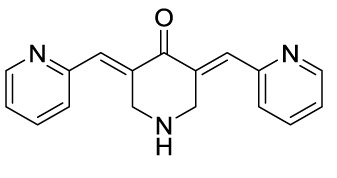	MSU-stimulated THP-1 cells and MSU-induced GA/Yes	Cathepsin B, NLRP3	(111)
NSC697923	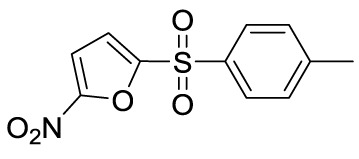	MSU-stimulated macrophages and MSU-induced GA/Yes	Caspase-1, NLRP3	(112)
MHC	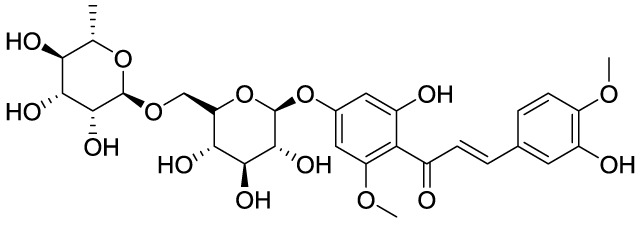	MSU-induced GA/Yes	NLRP3, ASC, pro-caspase-1, pro-IL-1β, Nrf2/HO-1, NF-κB	(113)
Spirodalesol analog 8A	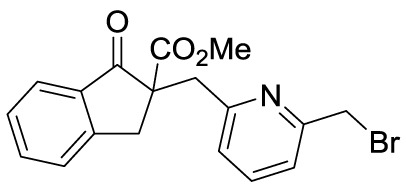	LPS-stimulated macrophages and MSU-induced GA/Yes	ASC, caspase-1,NLRP3	(114)
Z1456467176	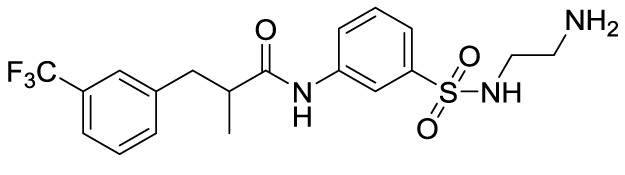	ATP-stimulated macrophages and MSU-induced GA/Yes	P2X7R, NLRP3, IL-1β	(115)

### Effect and mechanism of ncRNAs in the treatment of GA

**Figure 3:**
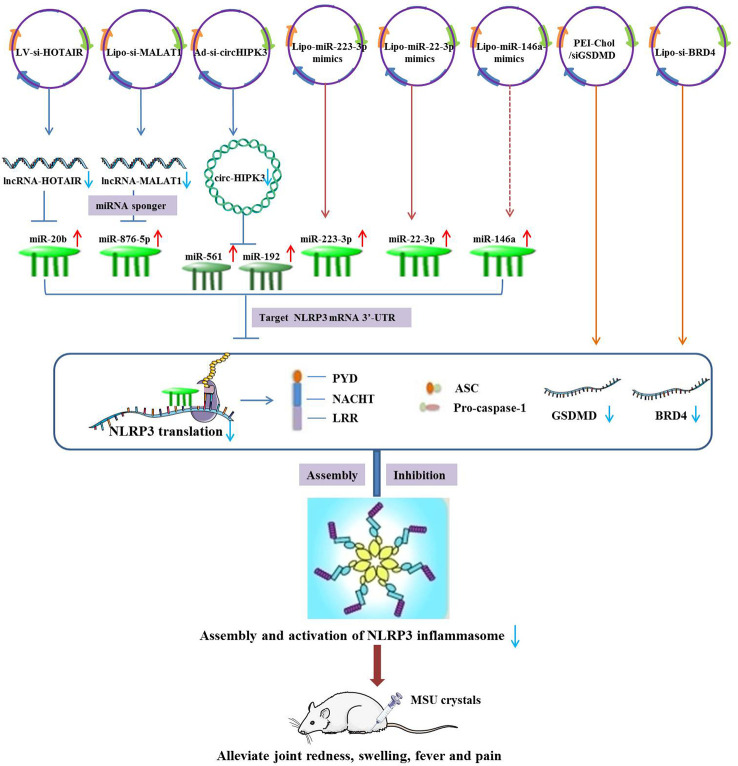
Therapeutic strategies of ncRNAs targeting NLRP3 in the treatment of GA.

Experimental study has suggested that the expression of lncRNA-HOTAIR and NLRP3 was up-regulated and miR-20b was down-regulated in synovial fluid mononuclear cells (SFMCs) derived from GA patients and MSU-stimulated THP-1 cells. Mechanistically, lncRNA-HOTAIR knockdown suppressed ankle swelling and inflammatory cytokine secretion in a mouse model of GA by sponging miR-20b and subsequently down-regulating NLRP3 expression (117). The expression of lncRNA-MALAT1 and NLRP3 were observed to be up-regulated in MSU-induced THP-1 macrophages. Silencing of MALAT1 by si-MALAT1 exerted a suppressive effect on MSU-induced inflammatory response through the negative regulation of MALAT1/miR-876-5p/NLRP3 axis (87). In addition, Lian et al. revealed that the elevated circRNA-HIPK3 in SFMCs may selectively sponge miR-192 and miR-561 to promote TLR4 and NLRP3 expressions, thereby boosting inflammatory response in GA. It was also demonstrated that circHIPK3 knockdown could significantly alleviate GA through the inhibition of NLRP3-mediated inflammatory signaling pathways and inflammatory cytokines levels (118). In a recent study, down-regulation of miR-223-3p and up-regulation of NLRP3 were detected in MSU-induced GA rats and FLSs. An encouraging finding is that overexpression of miR-223-3p could inhibit the inflammation and pyroptosis of MSU-induced FLSs and enhance cell viability by specifically targeting NLRP3, which may be served as a prospective biomarkers for the treatment of GA (119). Another study suggested that expression levels of miR-223-3p and miR-22-3p were significantly lower in both mice air pouch synovium and MSU-stimulated THP-1 cells than those in controls. Overexpression of miR-223-3p and miR-22-3p has been shown to diminish inflammatory response of GA by suppressing the assembly and activation of NLRP3 inflammasome, as well as the release of inflammatory cytokines (120). Zhang et al. observed that deficiency of miR-146a may promote more severe MSU-induced GA symptoms, inflammatory cytokine production and NALP3 inflammasome activation. Hence, overexpression of miR-146a was naturally thought to alleviate GA symptoms by degrading NALP3 (121). Unfortunately, this hypothesis has not been confirmed in the study. Gasdermin D (GSDMD) knockdown through siGSDMD-loaded PEI-Chol could effectively ameliorate ankle swelling, pyroptosis and inflammatory cascades in MSU-induced GA animal models by inhibiting NLRP3 inflammasome activation (122). *In vivo and vitro*, pretreatment of siBRD4 could attenuate joint swelling and synovial inflammation in MSU-stimulated THP-1 cells or GA rats by suppressing pyroptosis and NF-κB signaling, as well as NLRP3 inflammasome activation (123) (**Figure 3**).

## Conclusions and future perspective

Gout arthritis (GA), historically referred to as “the unwalkable disease”, is a chronic progressive inflammatory arthritis with a high rate of comorbidity and disability that severely impairs patients’ quality of life. Despite significant progress in the understanding the pathogenesis and treatment of GA, the prevalence of GA is increasing and many patients have been misdiagnosed and mistreated, resulting in an alarming situation of GA diagnosis and therapy. Acute GA attacks should be treated with NSAIDs, colchicine, or corticosteroids, or a combination of two therapeutic drugs. Urate-lowering medications to reverse hyperuricaemia is the fundamental method for long-term effective management of GA. However, owing to evident contraindications, severe adverse reactions and poor therapeutic outcomes, the clinical application of anti-inflammatory and urate-lowering medications is relatively limited, accounting for poor medication compliance and high GA recurrence rate. Therefore, the urgency to understand the pathophysiology of GA and find more effective therapy options has become exceedingly pressing.

It has been commonly accepted that NLRP3 inflammasome activation is of particular relevance to the pathogenesis of GA. More importantly, there is growing evidence that pharmacological inhibition of NLRP3 can effectively suppress the inflammatory response in MSU-stimulated macrophages as well as alleviate the inflammation and pain of GA mouse, indicating a promising development prospect and enormous clinical application potential. However, it must be acknowledged that the development of potential therapeutic agents targeting NLRP3 appears to be limited to preclinical studies rather than more significant breakthroughs. To broaden the scope of research, we reviewed some potential therapeutic medications targeting NLRP3, including natural products, synthetic compounds, and ncRNAs, which may be developed as a promising avenue for the treatment of GA.

At present, natural products have been the most extensively studied in the treatment of GA, and some of them have shown promising therapeutic prospects. Based on the screening and experimental validation of natural products for GA therapy, they will eventually provide theoretical basis and practical reference for the development of GA medications. However, there are still several challenges and unresolved issues that limit the development of therapeutic drugs. Specifically, most of them seem to exhibit better effects on relieving GA symptoms such as pain and inflammation, rather than completely curing GA. In addition, although these natural products serve a dual function in suppressing the NLRP3 inflammasome and alleviating GA symptoms, it has not been shown that these natural products exert therapeutic effects on GA by specifically targeting NLRP3. On the one hand, natural products have limited specificity and need large therapeutic dosages, which are not conducive to the exploitation of drugs for clinical use. On the other hand, natural products are faced with many issues such as water solubility and adverse effects. As a result, we must rely on structural modifications, computer-aided design, novel medicine delivery technology and other technical advancements to optimize natural products. In conclusion, better utilization of these natural products is the proper path to lead development of innovative medicines. Novel compounds described in this paper were mainly obtained by structure-activity relationship (SAR) screening and optimization using natural products as lead compounds. Target-based computer-aided drug design technologies will greatly facilitate drug development. The purity of synthetic compounds is relatively high, and the required dosage of therapeutic drugs is modest, both of which are in accordance with the drugs standards. Due to the relatively complicated process routes, ideas and technologies are particularly important. In recent years, gene therapy has been dubbed the “third revolution” in medicine and pharmacy because of its ability to fundamentally prevent, cure, or ameliorate different hereditary disorders, lending fresh historical relevance to ncRNAs. Gene therapy agents are the least researched and most target-specific, and therefore demand further study. Once the efficacy of NLRP3-targeting gene therapy medicines has been experimentally validated, the stability, safety, and delivery efficiency of delivery vectors may be further refined, which may be the mainstream development direction. In the near future, gene therapy will eventually shine in the treatment of resistant diseases with the ongoing development of emerging biotechnologies and materials.

As we reviewed, these natural products have shown positive anti-GA effects. Of note, the protective effect against GA through the inhibition of NLRP3 has only been experimentally validated at the levels of MSU-stimulated macrophages and GA mice. Due to the existence of biological species differences, some natural products that have good anti-GA properties at the cellular and animal levels may have poor therapeutic effect in human body, necessitating much deeper study in the future. Another point to consider is that most studies only implied that the identified natural products have an inhibitory effect on NLRP3, and there is no clear evidence that they can directly and specifically inhibit NLRP3, which may be responsible for the poor efficacy of anti-GA and treatment differences in humans and animals. Taken together, these natural products represent a vast treasure trove awaiting logical and orderly exploitation, and they may be exploited as lead compounds to develop more favorable therapeutic medications, such as dual-target therapies. Regulation of NLRP3 by ncRNAs is also a promising therapeutic approach for GA. Elucidating the underlying regulatory mechanisms mediated by NLRP3 will be of significant importance for developing reliable biomarkers and therapeutic targets. Notably, studies on the regulatory mechanisms the therapeutic applications of ncRNAs in GA by targeting NLRP3 are particularly lacking, which may be a weak direction for understanding the role in the pathogenesis and treatment of GA. At the moment, we are conducting experimental studies on the role of NLRP3 modulated by ncRNAs in the pathogenesis of GA, as well as the application of ncRNAs-related gene therapy agents to silence NLRP3 in the treatment of GA mouse models. In addition, with the integration and induction of numerous studies on the inhibitory effect of newly synthesized compounds on NLRP3 and the ameliorative effect on GA, the discussion of SAR and the discovery of effective pharmacophore should be strengthened, which may provide reliable reference and clues for the subsequent compound synthesis. To summarize, we have reason to anticipate that there will be considerable advances in anti-GA medicines by directly targeting NLRP3 in the near future.

## Author contributions

J-QW, Y-RL and JL conceived the original idea. J-QW and Y-RL wrote main manuscript and accomplished the Figures and Tables. JL polished the manuscript. All authors contributed to the article and approved the submitted version. 
